# Salinity Stress in Strawberry (*Fragaria* × *ananassa* Duch.): Biological Intervention Strategies and Breeding Approaches for Salt-Tolerant Cultivars

**DOI:** 10.3390/plants15030432

**Published:** 2026-01-30

**Authors:** Kondylia Passa, Maria Gerakari, Maria Goufa, Eleni Tani, Vasileios Papasotiropoulos

**Affiliations:** 1 Department of Agriculture, University of Patras, Nea Ktiria, 30200 Messolonghi, Greece; k.passa@upatras.gr; 2 Laboratory of Plant Breeding & Biometry, Department of Crop Science, Agricultural University of Athens, Iera Odos 75, 11855 Athens, Greece; mgerakari@aua.gr (M.G.); marog@aua.gr (M.G.); etani@aua.gr (E.T.)

**Keywords:** strawberry, soil salinity, breeding, stress, tolerance, *Fragaria*, biostimulants

## Abstract

Soil salinity is a major constraint to strawberry cultivation, adversely affecting plant growth, yield, and fruit quality. Salinity stress triggers complex physiological and biochemical responses, including osmotic adjustment, antioxidant defense, ion homeostasis, and shifts in metabolite accumulation, ultimately reducing crop productivity and fruit quality. This review synthesizes current knowledge on strawberry responses to salinity, with emphasis on physiological mechanisms, genotypic variation in tolerance, and emerging biologically based approaches, such as biostimulants, small signaling molecules, and beneficial microorganisms, that alleviate salinity stress and enhance plant resilience. In parallel, recent advances in the identification of stress-responsive genes and regulatory pathways are discussed in the context of their relevance for breeding salt-resilient cultivars. This review also identifies critical gaps in current knowledge that, despite significant progress, hinder the translation of mechanistic insights into stable yield and fruit quality under field conditions. By integrating physiological knowledge with advances in biological and breeding-based approaches, together with long-term field validation, this review provides a comprehensive framework for improving strawberry performance under saline conditions and guiding future cultivation and genetic improvement strategies.

## 1. Introduction

Agriculture, as the backbone of global food production, faces numerous challenges that threaten its sustainability and capacity to meet the nutritional demands of a growing population. Among these challenges, soil salinity has emerged as one of the most pressing abiotic stresses hindering crop productivity worldwide. Soil salinization, defined as the accumulation of soluble salts in the soil profile, represents a major form of land degradation with profound consequences for agriculture, particularly in terms of plant growth, performance, yield, soil health, and long-term sustainability [1,2].

Soil salinity encompasses saline, sodic, and alkaline soil types, each characterized by variable concentrations of salts and exchangeable cations. Saline soils contain high concentrations of soluble salts that reduce soil osmotic potential and impair plant water uptake, whereas sodic soils are dominated by sodium ions (Na^+^), leading to soil structural degradation and restricted water movement [1]. Alkaline soils, often associated with carbonate accumulation, exhibit high pH values that reduce nutrient availability and hinder root development [3]. Overall, excessive salts in the rhizosphere disrupt plant–soil–water interactions, adversely affecting crop growth, yield, and quality.

The magnitude of soil salinity as a global agricultural challenge is striking. According to recent estimates by the Food and Agriculture Organization (F.A.O.) [4], approximately 424 million hectares of surface soils and 833 million hectares of subsoils are affected by salinity worldwide. Salinity mainly affects riparian, semi-arid, and arid areas [5,6].

Mediterranean countries, including Greece, are particularly vulnerable to salinity due to their semi-arid climate, dependence on irrigation, and overexploitation of groundwater resources [7,8]. Climatic projections for the region indicate rising temperatures, reduced precipitation, and increased evapotranspiration, largely driven by climate change. These trends are expected to accelerate salt accumulation in irrigated soils, ultimately destabilizing yields and compromising crop quality [9,10,11].

The impact of salinity stress on plants depends on species and variety, growth stage, environmental conditions, and salt composition [12]. A comprehensive understanding of the physiological, biochemical, and molecular mechanisms governing plant stress responses is therefore essential for the development of effective mitigation strategies, including improved management practices and the breeding and cultivation of stress-resilient crop varieties [13,14]. Evidence from developed countries indicates that the adoption of sustainable cultivation practices combined with salinity-tolerant genotypes has enabled the restoration and productive use of salt-affected soils, whereas in many developing regions, reliance on short-term management approaches has failed to provide lasting solutions, thereby exacerbating food insecurity [15].

## 2. Salinity Stress as a Constraint in Strawberry Cultivation

### 2.1. Impacts of Salinity Stress on Strawberry

Strawberry represents a particularly important case study for salinity tolerance, due to its high economic value, widespread cultivation, and pronounced sensitivity to soil and water salinity [16,17].

Belonging to the Rosaceae family, strawberries are prized for their unique flavor, nutritional value, and bioactive compounds, including anthocyanins, polyphenols, vitamins, and minerals, which contribute to their recognized health-promoting properties [18,19].

In response to strong market demand, global strawberry production has expanded over the past decade into arid and saline regions where irrigation water quality is often poor [20]. Therefore, secondary salinization has become increasingly prevalent, exposing strawberries to elevated salt stress. Strawberry plants are glycophytes and display a low tolerance to salinity, with yield reductions reported at soil electrical conductivity (ECe) values as low as 1.0 dS m^−1^ [17]. For each additional unit increase in ECe, yield declines by approximately 33%, making salinity one of the most severe constraints on strawberry cultivation in irrigated regions [21].

Research on strawberry salinity tolerance has expanded in recent years, addressing genotypic differences as well as the morphological, physiological, biochemical, and molecular responses underlying stress adaptation [22,23,24,25,26,27,28]. Focused on the effects of salinity on fruit quality and yield, strawberry research highlights significant physiological and metabolic adjustments that influence consumer-relevant traits [17,29,30].

Based on research findings, secondary salinization induces pronounced physiological stress in strawberry plants [31,32]. Elevated salt concentrations reduce water uptake through osmotic effects and promote the accumulation of sodium and chloride ions in plant tissues, where they compete with essential nutrients such as potassium and calcium [33]. This ionic imbalance disrupts photosynthesis, stomatal regulation, and enzyme activity. Salt stress also enhances the production of reactive oxygen species (ROS), leading to oxidative damage of cellular membranes and proteins. Although antioxidant defense mechanisms are present, their capacity is often insufficient under prolonged salinity, resulting in leaf chlorosis, growth inhibition, and reduced yield.

Salinity stress affects not only productivity but also fruit quality by altering the balance of sugars and organic acids and influencing the accumulation of secondary metabolites, which are critical determinants of fruit flavor and nutritional value. As a result, key quality traits such as sweetness, acidity, firmness, shelf life, and nutritional attributes are compromised, ultimately reducing market value and consumer acceptability [17,29,34].

**Figure 1:**
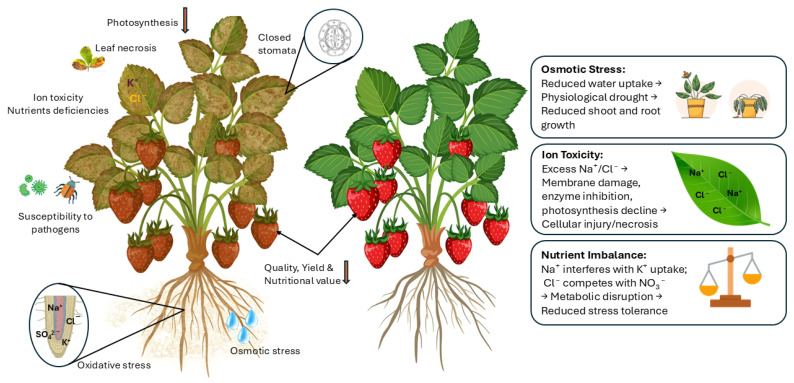
Overview of the physiological and agronomic implications of salt stress in strawberry plants.

An overview of the effects of salt stress on strawberry growth, development, and productivity is provided in Figure 1.

The severity of yield and quality losses can escalate when salinity is combined with shallow groundwater and waterlogging, which together intensify stress conditions and limit root aeration [31,32]. For strawberry cultivation, strategies such as soil flushing, adoption of salt-tolerant cultivars, and precision irrigation techniques can help mitigate stress and sustain production.

Salinity stress negatively affects strawberry growth, yield, and fruit quality through a range of physiological and metabolic disturbances. The extent of these effects varies depending on plant material and management conditions. Improving salinity tolerance therefore relies on the identification and development of tolerant cultivars, alongside the adoption of management practices that support soil and water sustainability. A clearer understanding of strawberry responses to salinity can contribute to the development of more resilient production systems under increasingly challenging environmental conditions.

The aim of this review is to provide a critical synthesis of physiological, biochemical, and molecular mechanisms, biological interventions, and breeding-based approaches for improving strawberry tolerance to salinity stress, including biostimulants, stress-related genes, and existing variation among strawberry cultivars and crop wild relatives. This review identifies key knowledge gaps and research priorities to support strawberry cultivation and genetic improvement under saline conditions.

### 2.2. Variation in Salinity Responses Across Different Strawberry Species and Cultivars

Strawberry species and their wild ancestors exhibit distinct responses to salinity stress, largely due to differences in genetic background and genome complexity. The genus *Fragaria* comprises 16 priority crop wild relatives (CWRs) that can be exploited in plant breeding to enhance cultivated strawberries, offering opportunities to improve fruit quality, tolerance to abiotic stresses, and resistance to pests and diseases [35]. Comparative analyses of salinity tolerance mechanisms across *Fragaria* species are therefore essential for identifying key regulatory nodes and molecular pathways underpinning adaptive responses to salinity stress.

Among these CWRs, the Chilean strawberry (*F. chiloensis* Duch.), an octoploid wild ancestor of the cultivated strawberry comprises four subspecies: *F. chiloensis* subsp. *chiloensis*, subsp. *lucida*, subsp. *pacifica*, and subsp. *sandwicensis*. This species is widely regarded as tolerant to abiotic stresses, particularly salinity and drought, and thus represents a valuable genetic resource for strawberry breeding [36,37,38]. Consistent with this view, Nikoloudi [39] demonstrated pronounced differences in salinity stress responses among *Fragaria* CWRs, showing that although *F. virginiana* and *F. chiloensis* are wild progenitors of the cultivated strawberry, *F. chiloensis* exhibits a substantially greater capacity to withstand saline conditions.

Subsequent comparative studies involving cultivated varieties and CWRs further substantiated these findings. Garriga et al. [40] evaluated salinity effects on growth performance, physiological responses, productivity, and fruit quality in three strawberry genotypes: the commercial cultivar ‘Camarosa’, the cultivated Chilean strawberry ‘Bau’ (*F. chiloensis* subsp. *chiloensis* f. *chiloensis*), and the wild Chilean strawberry ‘Cucao’ (*F. chiloensis* subsp. *chiloensis* f. *patagonica*). Notably, *F. chiloensis* f. *patagonica* was largely unaffected by salt stress regarding yield losses, indicating superior salinity tolerance among the genotypes examined.

Towards this end, Kortekamp et al. [41] assessed salinity tolerance in an F_2_ population derived from a cross between the salt-sensitive cultivar ‘Senga Sengana’ and a salt-tolerant *F. chiloensis* ssp. *lucida* accession. Under greenhouse salt stress treatments, F_2_ individuals exhibited variable necrotic damage and growth reduction, revealing genetic variation in salt tolerance and identifying genotypes that could serve as a valuable starting material for breeding strawberries with enhanced stress resilience.

These findings underscore the importance of exploiting CWR diversity in strawberry improvement programs, as many researchers have emphasized that the incorporation of resilient wild genotypes into breeding strategies is essential for the development of new varieties capable of minimizing yield losses in saline soils.

A wide range of strawberry commercial cultivars has been evaluated for tolerance to salinity stress [20,22,23,25,29,33,41,42,43,44,45,46,47,48,49,50,51,52,53,54]. Nevertheless, only a limited number of studies have explicitly investigated the relationship between salinity tolerance and yield penalties (Table 1).

Most studies focus on the response of strawberry cultivars to salt stress through the assessment of agronomic, physiological, and biochemical traits, often evaluated before plants reach the fruiting stage. Accordingly, vegetative growth is commonly used as an early indicator of salt sensitivity, as it is generally faster and, in many cases, more reliable than yield-related traits. Supplementary Table S1 summarizes the most representative studies.

Heterogeneity in experimental design, including differences in NaCl concentrations, methods of salinity imposition (acute stress versus gradual increases in NaCl doses), and the duration of stress treatments, has hindered the ability to draw definitive conclusions. For instance, ‘Camarosa’ is often reported to be more tolerant than other cultivars under mild salinity conditions [47,54]. Nevertheless, substantial yield reductions have been documented in other studies [22,50], indicating that its response is strongly influenced by the cultivation system (hydroponics versus soil) and by the manner in which salt stress is imposed, including practices such as intermediate leaching.

Studies that have explicitly monitored yield responses have consistently reported substantial yield reductions, even in cultivars classified as relatively tolerant. For example, Ferreira et al. [17] reported significant yield reductions in the cultivar ‘Albion’ under moderate salinity (2.5 dS m^−1^) in field experiments, although it was classified as the cultivar exhibiting the lowest reductions in fruit yield, commercial fruit size, and plant survival. Similarly, under elevated salt stress (80 mmol NaCl L^−1^), Keutgen and Pawelzik [33] observed fresh weight reductions of 26% in the ‘tolerant’ cultivar ‘Korona’ in greenhouse experiments. Denaxa et al. [22] also documented yield losses in the relatively tolerant cv. ‘Rociera’, with average reductions of 17.3% and 22.7% under moderate and high saline conditions (2 dS m^−1^ or 4 dS m^−1^, respectively) in greenhouse experiments.

Collectively, these findings highlight considerable variability in the magnitude of yield loss among cultivars under saline conditions; however, they also clearly demonstrate that no cultivar is exempt from yield penalties under salinity stress. The genetic material (both wild and cultivated) exhibiting the lowest yield losses under salinity treatments is summarized in Table 1.

Interestingly, it has been reported by Alnayef et al. [48] that mild salinity (10 mM) can be utilized to improve fruit quality by increasing antioxidant capacity and phenolic accumulation in ‘Elsanta’ and ‘Elsinore’, with a relatively low impact on yield (approximately 14–16% loss) when combined with appropriate fertilization. A finding also observed by Galli et al. [20], who reported that mild salt stress did not affect fruit yield in ‘Camarosa’, while low salt levels enhanced photosynthesis, vegetative growth, and anthocyanin and sucrose contents. Therefore, application of mild salt stress may be an effective biofortification strategy.

Taken together, these studies underscore that improving salt tolerance while preserving yield stability and fruit quality should be a central objective of strawberry breeding programs. However, the availability and identification of genetic material combining these traits remain limited, highlighting a critical gap in the current literature and the availability of breeding resources. Traits such as efficient ionic regulation, enhanced antioxidant capacity, osmotic adjustment, modified stomatal behavior, the ability to balance Na^+^ exclusion with Cl^−^ compartmentalization, and the maintenance of relative water content under moderate salinity are widely considered important determinants of salinity tolerance. However, effective breeding for saline environments must also prioritize yield stability and the preservation of fruit quality traits, as physiological tolerance alone does not necessarily prevent yield or quality penalties under stress conditions.

## 3. Mitigation Strategies to Enhance Strawberry Performance Under Salinity Stress

Salinity stress represents a significant and growing challenge for strawberry cultivation worldwide. In response, a range of mitigation strategies has been explored, including the use of various biostimulants and other exogenous signaling compounds that modulate plant physiological and molecular stress responses, practices related to supplemental lighting, and the exploitation of beneficial microorganisms and plant–microbe interactions that enhance nutrient acquisition, ion homeostasis, and structural resilience. A deeper understanding of the mechanisms and effects of these complementary approaches is essential for supporting resilient and sustainable strawberry production under increasingly saline and environmentally challenging conditions.

### 3.1. The Role of Biostimulants and Small Signaling Molecules in Mitigating Salinity Stress

The external application of classical biostimulants and of small signaling molecules has been shown to significantly mitigate the adverse effects of various abiotic stresses, including salinity [55]. Compounds such as 5-aminolevulinic acid (ALA), melatonin (N-acetyl-5-methoxy-tryptamine) (MEL), salicylic acid (SA), humic acid (HA), acetic acid, methyl jasmonate (MeJA), zinc oxide (ZnO) and silicon nanoparticles (NPs), γ-aminobutyric acid (GABA), and related substances have been investigated for their ability to alleviate salinity-induced damage and enhance stress tolerance in strawberry [56,57,58,59,60,61].

ALA, a precursor of tetrapyrroles including chlorophyll, phytochrome, and heme, has been shown to enhance photosynthetic efficiency and antioxidant defenses at *F. × ananassa*, cv. Benihonpe plants. Root-applied ALA improves PSII and PSI activity, sustains electron transport, reduces photo-inhibition, and promotes osmotic adjustment under both NaCl-induced salinity (100 mM) and osmotic stress (PEG) in experiments conducted under greenhouse and controlled growth chamber conditions, respectively [62,63]. At the molecular level, ALA modulates stress-responsive genes and promotes H_2_O_2_-mediated signaling that enhances root Na^+^ retention while limiting leaf Na^+^ accumulation [63]. Evidence suggests that 10 mg L^−1^ ALA is generally effective, although optimization may depend on developmental stage and environmental conditions [64]. Despite these benefits, direct effects of ALA application on fruit yield remain largely unexplored.

Furthermore, MEL is another important small signaling molecule that plays a vital role as an antioxidant in plant stress responses. Under salt stress, MEL usually accumulates in plant tissues, functioning as a ROS scavenger and enhancing both enzymatic antioxidant capacity and the maintenance of photosynthetic pigments [60,65,66,67]. The application of MEL in strawberry plants remains poorly documented. In a greenhouse experiment, foliar application of MEL at concentrations of approximately 100–200 μM in ’Camarosa’ was associated with improvements in fruit yield and quality under moderate to high NaCl stress (40–80 mM), including higher soluble solids, ascorbic acid, total antioxidant capacity, phenolic content, and sugar levels. MEL has also been suggested to modulate abscisic acid (ABA) signaling, potentially contributing to enhanced stress resilience [68]. However, the underlying metabolic and regulatory responses, as well as the consistency and extent of yield-related effects in strawberries, remain insufficiently characterized, highlighting the need for further investigation.

Moreover, SA, a phenolic signaling molecule, has been found to improve growth, chlorophyll content, water status, nutrient uptake, and antioxidant defenses in strawberry under salinity stress [69]. On the strawberry cultivars ‘Camarosa’, ‘Selva’, and ‘Gaviota’, pot experiments conducted under greenhouse and field conditions have shown that both foliar and pre-treatment applications can mitigate oxidative stress and help maintain physiological performance under salinity. The timing of application appears to be critical, with pre-treatment prior to salt exposure often associated with more pronounced beneficial effects. Based on the existing literature, an optimal combination of SA and salinity stress for enhancing antioxidant capacity, photosynthetic performance, nutrient retention, and, importantly, fruit yield and quality is approximately 0.5–0.9 mM (30–90 ppm) SA, depending on cultivar and growth stage, combined with moderate salinity (~40 mM NaCl). Foliar application one week before stress onset is generally recommended for best growth and physiological outcomes [70,71,72,73,74]. However, while several studies report improvements in photosynthetic performance and stress-related indices, only a limited number have demonstrated tangible increases in fruit yield and quality.

In addition, exogenous GABA application has been shown to enhance strawberry tolerance to salinity by coordinating antioxidant defense, osmotic adjustment, and photosynthetic protection. Transcriptomic analyses showed that foliar GABA (0–20 mM) upregulated chlorophyll metabolism and photosynthesis-related genes, with WRKY, AP2/ERF, and MYB transcription factors contributing to stress resilience [75]. Complementarily, a similar study demonstrated that 25 mM GABA reduced ROS and MDA, improved membrane stability, and increased expression of stress-responsive genes (*DREB*, *cAPX*, *MnSOD*, *GST*), also acting as a priming signal under non-stress conditions [76]. Together, these findings indicate that GABA functions as both a rapid protective agent and a transcriptional regulator, highlighting its potential for breeding salinity-resilient strawberry varieties.

HA has also been reported to confer beneficial effects under salinity stress in various plant species by improving soil physicochemical properties, as well as by enhancing plant morphological traits, physiological processes, and overall biochemical responses [77,78,79]. To date, evidence for HA application in strawberry is limited to a single study conducted in Iran on the cultivars ‘Kurdistan’ and ‘Paros’, in which root application of HA was associated with reduced Na^+^ accumulation, enhanced K^+^ uptake, improved photosynthetic performance, and partial recovery of growth and yield. Importantly, the experiments were carried out over multiple seasons, and the authors reported consistent beneficial effects across cultivars [80]. Nevertheless, comprehensive field-scale validation and direct evidence linking HA application to stable commercial yield improvements in strawberry remain limited.

Furthermore, in hydroponic greenhouse experiments, foliar spraying of exogenous acetic acid (1–2 mM) [81] and foliar or nutrient solution applications of MeJA and silicon- NPs [82] have been shown to improve salt tolerance in the strawberry cultivar ‘Paros’ by enhancing antioxidant defenses, promoting osmolyte accumulation, maintaining photosynthetic performance, and regulating stress-responsive gene expression under moderate salinity (40–50 mM NaCl). These compounds may act synergistically to mitigate oxidative damage and preserve membrane stability, highlighting their potential for combined biostimulant strategies.

Complementary evidence from controlled in vitro experiments indicates that ZnO NPs can also alleviate salinity stress in strawberries. Zeid et al. [83] reported cultivar-dependent responses in *F. × ananassa*, with moderate ZnO-NP applications reducing Na^+^ accumulation, improving K^+^ uptake, and enhancing antioxidant activity under NaCl stress. However, whether these nanoparticle-mediated effects translate into improved whole-plant performance under field conditions remains to be established. Small signaling molecules and biostimulants can serve to enhance strawberry tolerance to salinity by protecting photosynthesis, strengthening antioxidant defenses, maintaining osmotic and ion balance, and regulating stress-responsive genes. Root- or foliar-applied ALA, MEL, SA, HA, GABA, acetic acid, MeJA, and NPs consistently improve these physiological and molecular responses. Despite these beneficial findings, most studies focus on early vegetative growth or physiological and biochemical markers, with limited data on fruit yield, quality, or field performance. Treatment protocols vary widely, and large-scale field validations are scarce. Future research should prioritize standardized, long-term trials linking molecular responses to reproductive performance to fully realize the agronomic potential of these biostimulants.

### 3.2. Microorganisms and Plant–Microbe Interactions to Alleviate Salinity Stress

In recent years, there has been increasing interest in the role of beneficial soil microorganisms, particularly arbuscular mycorrhizal fungi (AMF), in enhancing plant tolerance to salinity. These symbiotic fungi interact with plant roots, facilitating improved water and nutrient acquisition, especially phosphorus and micronutrients, and modulating physiological responses under salt stress [84,85]. In a greenhouse experiment, inoculation of two-month-old strawberry plants (*F. × ananassa* cv. Tochiotome) with *Gigaspora margarita* under high salinity (200 mM NaCl) significantly improved shoot and root biomass, maintained chlorophyll content, and reduced leaf browning. AMF symbiosis also promotes ion homeostasis by reducing Na^+^ accumulation and decreasing the Na^+^/K^+^ ratio in both roots and shoots [86]. AMF appears to enhance both physiological performance and structural adaptations, providing a multifaceted protective mechanism for strawberry plants under salinity stress. However, further experimentation is required to confirm the beneficial effects of AMF on strawberry performance under salinity conditions, as well as to elucidate their potential impact on yield and yield-related traits.

Field- and greenhouse-based studies provide increasing evidence for the role of plant growth-promoting rhizobacteria (PGPR) in mitigating salinity stress in strawberries. Under naturally saline soil conditions, inoculation with halotolerant PGPR strains significantly improved plant performance in strawberry cultivation, resulting in enhanced vegetative growth, higher chlorophyll content, improved nutrient acquisition (including N, P, K, Ca, Mg, and micronutrients), reduced electrolyte leakage, increased leaf relative water content, and substantial yield gains, with some strains increasing fruit yield by approximately 46–48%; however, the effectiveness of such approaches is likely to depend on strain specificity, environmental conditions, and cultivar background [87]. Similarly, PGPR consortia isolated from saline environments have been shown to enhance biomass accumulation, photosynthetic performance, and overall stress tolerance under moderate salinity (85 mM NaCl), as well as under climate change–simulated conditions combining elevated CO_2_ and temperature, indicating potential relevance for future production systems for combined environmental stresses anticipated under climate change scenarios [87,88,89].

Further evidence suggests that PGPR effects may be amplified when combined with organic soil amendments. Under saline conditions, the joint application of PGPR with humic- and fulvic acid–based fertilizers resulted in marked improvements in strawberry fruit weight and total yield, with combined treatments generally outperforming organic amendments applied alone [88]. It has also been reported that in strawberry, the efficacy of PGPR under salinity depends more on strain–plant–environment compatibility than on microbial diversity per se, supporting the targeted use of native PGPR for improving crop productivity in saline environments [90]. These results indicate synergistic interactions between organic soil promoters and carefully selected PGPR, which not only enhance nutrient availability and root function but also translate into consistent yield gains and improved yield stability under salt stress.

Microbial and microbe-derived interventions enhance strawberry salinity tolerance by maintaining ion homeostasis, improving nutrient uptake, reinforcing root structure, boosting antioxidant defenses and photosynthesis, and activating stress-responsive genes and metabolic pathways. Despite these benefits, most studies focus on vegetative growth under controlled conditions, with limited data on fruit yield, quality, or long-term field performance. Furthermore, the interactions of salinity with other abiotic stresses and the detailed molecular mechanisms underlying microbe-mediated tolerance remain poorly understood. Future research should prioritize standardized, field-based trials that link physiological and molecular responses to reproductive performance and commercial yield to fully realize the agronomic potential of these strategies.

### 3.3. Supplemental Light Application

Supplemental lighting has been explored to modulate plant physiological responses to salinity stress. Recent studies demonstrate that manipulation of light spectral quality can effectively mitigate the adverse effects of salinity and alkalinity stress in strawberry by targeting photosynthetic and physiological processes [91,92,93]. Esmaeilizadeh et al. [91] investigated the effects of different spectra of supplemental light on strawberry (cv. Camarosa) under salt and alkalinity stress conditions. The authors demonstrated that red and red/blue light had a positive effect on CO_2_ assimilation, while blue/red light increased intrinsic water use efficiency (WUEi) under both stress conditions. Adjustment of light spectra, especially red light, increased photosystem II (PSII) performance indices and quantum yield parameters.

Moreover, Malekzadeh Shamsabad et al. [92] extended the analysis and showed that plants adopt different strategies against abiotic stress depending on light quality. Supplemental blue/red and white/yellow light partially restored vegetative growth, chlorophyll content, relative water content, and mineral nutrient balance, while limiting Na accumulation in plant tissues. Importantly, these light treatments increased the number of inflorescences and fruits and enhanced early fruiting, demonstrating that improvements in the performance of the photosynthetic apparatus can translate into improved reproductive output under stress conditions. More recent work by Malekzadeh Shamsabad et al. [93] further showed that spectral light improves not only early fruit yield but also fruit biochemical quality. Supplemental light spectra enhanced the accumulation of anthocyanins, total phenolic compounds, and antioxidant activity, while modulating titratable acidity in strawberry fruits.

Collectively, these studies provide a mechanistic framework linking light-regulated photosynthetic apparatus performance, ion homeostasis, and metabolic allocation to improved reproductive performance and fruit nutritional quality in strawberries cultivated under saline environments.

Finally, the application of hydrogen-rich water (HRW), a stress modifier, to strawberry seedlings under salinity stress enhances root biomass, promotes K^+^ uptake, strengthens antioxidant defenses, and reduces Na^+^ accumulation. Transcriptomic and metabolomic analyses indicate activation of genes and pathways involved in ion transport, antioxidant defense, and cell wall biosynthesis, with phenylpropanoid biosynthesis and amino/nucleoside sugar metabolism playing particularly important roles in salt stress mitigation [94].

**Figure 2:**
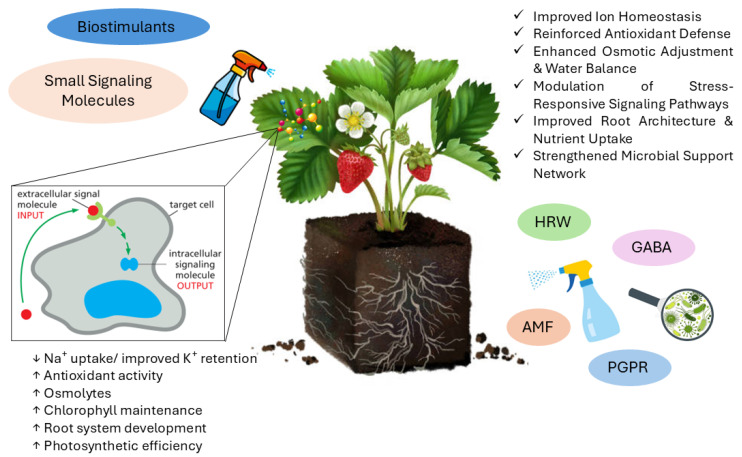
Overview of the beneficial effects through the exogenous application of small signaling molecules, biostimulants, and plant–microbe interactions (AMF: arbuscular mycorrhizal fungi; PGPR: plant growth-promoting rhizobacteria; HRW: hydrogen-rich water; GABA: γ-aminobutyric acid) in enhancing salinity tolerance in strawberry.

The roles of biostimulants, signaling molecules, HRW, and plant–microbe interactions in enhancing salinity tolerance are shown in Figure 2.

The above-discussed research findings of Section 3 are presented in detail in Supplementary Table S2, including information on the experimental setting, plant material and developmental stage, growth conditions, salinity stress level and duration, and parameters analyzed.

## 4. Genetic and Molecular Approaches for Improving Salinity Tolerance in Strawberry

### 4.1. Gene Identification and Functional Annotation for Breeding Targets

Salinity tolerance in strawberry involves the coordinated regulation of ion homeostasis, osmotic adjustment, oxidative stress detoxification, and transcriptional control, as evidenced by recent physiological and molecular studies. Gene identification and functional annotation provide a critical framework for understanding the molecular mechanisms underlying complex stress-adaptive traits, enabling the discovery of candidate genes that can be targeted to enhance stress tolerance through breeding [95]. However, consistent with broader trends in crop salinity research, most strawberry studies assess tolerance primarily through physiological, biochemical, and transcriptional responses associated with vegetative growth and survival, whereas direct evidence linking these mechanisms to fruit yield and quality remains limited [96,97]. To enable systematic comparison across studies, stress-responsive genes reported in the literature were organized into major functional categories reflecting their roles in key processes underlying salinity tolerance. The genes were grouped according to their primary molecular functions, while regulatory components such as transcription factors and signaling proteins may contribute to multiple physiological stress-response pathways (Table 2). This framework facilitates evaluation of their contributions to stress resilience and relevance for breeding. In addition, the experimental settings, plant developmental stages, key growth conditions, and salinity stress protocols used across all studies summarized in Table 2 are compiled in Supplementary Table S3.

#### 4.1.1. Ion Homeostasis- and Transport-Related Genes

Among the functional categories, ion homeostasis- and transport-related genes show the clearest association between molecular responses and agronomic performance. In strawberry, key genes in Na^+^ (*SOS1*, *SOS2*, *SOS3*, *NHX1/NHX2*) and Cl^−^ (*CLC*-G, *CLC*-C) transport are consistently associated with reduced ionic toxicity, improved ion partitioning, and superior whole-plant performance under saline irrigation [98,100,112]. Importantly, these associations are supported by experimental studies conducted directly in strawberry, including continuous saline irrigation under greenhouse conditions, where genotypic differences in Cl^−^ accumulation and partitioning correlate strongly with biomass maintenance and stress tolerance indices [98].

The relevance of these findings is reinforced by evidence from rice, where functional alleles of *OsSOS1* and *OsNHX1* contribute to improved biomass and grain yield under moderate salinity by limiting Na^+^ accumulation in photosynthetically active tissues [96,97]. The consistency between strawberry-based physiological evidence and yield-validated rice examples supports the conclusion that strawberry orthologs of *SOS* and *NHX* genes represent realistic breeding targets, particularly when selection focuses on ion exclusion and compartmentation phenotypes.

In addition, several transcriptional regulators are functionally linked to ion homeostasis in strawberry, including FvMYB24 and FaTEDT1L, for which functional assays—often involving heterologous expression of the corresponding genes—indicate activation of conserved ion-regulatory pathways and enhanced salt tolerance [99,100]. FvMYB24, an R2R3-MYB transcription factor from *F. vesca*, conferred enhanced salinity tolerance when overexpressed in *Arabidopsis thaliana*, as evidenced by improved germination, root elongation, fresh biomass, and chlorophyll retention under salt stress [99]. At the molecular level, FvMYB24 is directly bound to the promoter of *SOS1* and positively regulates the expression of key salt-responsive genes, including *SOS1*, *NHX1*, and *LEA3*, thereby linking transcriptional control to Na^+^ transport and ion homeostasis. Comparable regulatory behavior was observed for the HD-ZIP IV transcription factor FaTEDT1L, whose overexpression in transgenic Arabidopsis enhanced salt tolerance via increased root growth, reduced water loss, and activation of SOS pathway genes (*SOS1*, *SOS2*, *SOS3*) [100]. Despite this clear mechanistic role, these findings remain largely derived from heterologous systems and early developmental stages, limiting direct inference regarding fruit yield or long-term growth performance in strawberries. Accordingly, their breeding utility is likely to depend on allelic variation or context-specific regulation rather than constitutive expression, given that transcription factors controlling ionic responses may introduce growth penalties when misregulated [118,119].

#### 4.1.2. Osmotic Adjustment and Water Balance

Osmotic adjustment and water balance constitute a central component of strawberry salinity responses (Table 2). The accumulation of osmotin (PR-5) under low water potential represents one of the earliest documented osmoprotective mechanisms and has been demonstrated directly in strawberry tissues [101,102]. More recent studies identify transcription factors such as FaNAC2, FvNAC29, FaTINY2 (AP2/ERF), and FaTEDT1L (HD-ZIP IV) as regulators of proline accumulation, ABA signaling, and transpiration control [100,103,104,105].

For many of these regulatory genes, functional validation relies heavily on heterologous expression in *Arabidopsis*, where altered survival, root growth, and water-loss phenotypes provide strong mechanistic support. For example, FaNAC2 from ‘Benihoppe’ strawberry, a member of the ATAF subfamily, was shown to be induced by multiple abiotic stresses and, when overexpressed in *Nicotiana benthamiana*, enhanced salt, cold, and drought tolerance through upregulation of proline and ABA biosynthesis pathways [103].

Additional evidence comes from transgenic approaches in strawberries. Husaini et al. [102] reported that plantlets overexpressing osmotin displayed enhanced salt tolerance in vitro, including higher proline and soluble protein accumulation, improved chlorophyll retention, and survival under NaCl concentrations up to 150 mM. However, these lines also exhibited slightly reduced growth under non-stress conditions, indicating a potential fitness cost associated with constitutive overexpression. Crucially, it remains unknown whether elevated osmotin expression translates into yield or fruit-quality benefits under greenhouse or field conditions.

Similarly, heterologous expression of the woodland strawberry gene *FvNAC29* in Arabidopsis resulted in reduced ROS and malondialdehyde accumulation, increased proline and chlorophyll content, and enhanced activities of antioxidant enzymes (CAT, SOD, POD), indicating coordinated regulation of osmoprotective and oxidative stress responses under salinity [104]. FaTEDT1L has been associated with reduced water loss and enhanced root growth under salinity in transgenic assays, suggesting a contribution to water-balance regulation alongside its effects on ionic pathways [100].

In addition, overexpression of the AP2/ERF transcription factor FaTINY2 from octoploid strawberry was associated with enhanced antioxidant defense, osmotic adjustment, and reduced oxidative damage, as reflected by elevated proline and chlorophyll levels and increased CAT, SOD, and POD activities in transgenic lines [105,120]. Even so, such assays do not capture strawberries’ perennial habit or the way resources are partitioned between leaves, roots, and developing fruits. Consistent with observations in rice and wheat—where NAC and AP2/ERF transcription factors enhance drought or salt tolerance but exhibit variable yield effects—osmotic adjustment genes in strawberry should currently be regarded as contributors to stress resilience rather than validated yield-stability determinants [119,120].

#### 4.1.3. Oxidative Stress Regulation and Redox Balance

Salinity-induced oxidative stress is consistently observed in strawberry, with enhanced activity of SODs (FaCSDs, FaMSDs), catalases, peroxidases, and increased NADPH production via FaG6PDHs contributing to reduced ROS accumulation and membrane damage [106,107]. These responses are measured directly in strawberry tissues and correlate with improved physiological performance under salt stress. At the genetic level, genome-wide identification and expression analyses of the G6PDH family suggest that multiple members contribute to redox homeostasis through enhanced NADPH production under salinity [107]. Further support for the role of antioxidant systems in strawberry comes from a targeted analysis of *superoxide dismutase* (*SOD*) genes in octoploid cv. Benihoppe. Zhang et al. [106] identified salinity-induced expression of specific isoforms, including FaMSOD5, highlighting their contribution to ROS detoxification under salt stress. The study also revealed distinct expression patterns of several *FaCSD* genes (e.g., *FaCSD1*, *FaCSD2*, *FaCSD7*, *FaCSD10*) across fruit ripening stages. Because ROS function both as damaging agents under salt stress and as signaling molecules during fruit maturation, coordinated SOD activity may indirectly support yield stability by maintaining vegetative performance and normal fruit development. However, functional validation under greenhouse or field conditions is still necessary to confirm the agronomic relevance of these candidates.

In addition, the MYB transcription factor FvMYB82 (Section 4.1.4) was associated with enhanced antioxidant capacity under salinity, as indicated by increased activities of SOD, POD, and CAT in transgenic lines [112].

Regulatory genes such as *FaZAT10* and *MYB5*, which modulate antioxidant and flavonoid biosynthesis pathways, are largely validated through heterologous expression systems, where improved oxidative stress tolerance is readily observed [108,109]. In cultivar-level comparisons, MYB5-associated anthocyanin accumulation has been shown to track with reduced oxidative damage and improved physiological performance under salinity, reinforcing its role as a modulator of antioxidant capacity rather than a direct yield determinant [109]. Evidence from rice and tomato indicates that antioxidant capacity can support yield maintenance under salinity only when ionic stress is effectively controlled [97]. Accordingly, oxidative stress-related genes in strawberry are best interpreted as supportive components of tolerance rather than primary breeding targets.

#### 4.1.4. Transcriptional Regulators

A substantial proportion of strawberry salinity research focuses on transcription factors, including NAC, MYB, AP2/ERF, GRF, HD-ZIP, and zinc-finger families (Table 2) [99,100,104,105,108,109,110,111,112]. These regulators integrate multiple stress-response pathways and therefore provide strong mechanistic entry points for understanding salinity adaptation. Consistent with this integrative role, several transcription factors discussed here also appear in ion homeostasis and osmotic adjustment sections, where their downstream physiological effects are highlighted.

For example, Li et al. [111] characterized ten *FvGRFs* in *F. vesca*, grouped into five subfamilies based on conserved QLQ and WRC domains linked to chromatin remodeling and transcriptional control. Among them, *FvGRF3*, *FvGRF6*, and *FvGRF8* showed notable salt-induced upregulation, with *FvGRF8* displaying broader tissue expression including fruit, suggesting potential roles in both stress signaling and fruit physiology.

Functional characterization of the MYB transcription factor family has revealed that FvMYB82 from *F. vesca,* through coordinated regulation of osmotic and oxidative stress responses, enables transgenic lines expressing *FvMYB82* to accumulate higher proline and chlorophyll and exhibit elevated activities of key antioxidant enzymes (SOD, POD, CAT), consistent with coupling of osmoprotective mechanisms to improved redox homeostasis under salt stress [112]. Moreover, FvMYB24 has already been analyzed in Section 4.1.1. for its role in ion homeostasis.

The HD-ZIP IV factor FaTEDT1L has been reported to enhance salt tolerance in transgenic assays, with phenotypes including improved root growth, reduced water loss, and induction of *SOS* pathway genes (*SOS1–SOS3*). These results suggest that FaTEDT1L can couple water-balance regulation with upstream transcriptional modulation of ionic stress responses, although direct validation of ionic outcomes and yield effects in strawberry remains limited [100].

More broadly, these examples indicate that functional evidence for many strawberry transcriptional regulators is derived largely from heterologous expression systems and assays conducted at early developmental stages. While such approaches provide strong mechanistic support, they cannot reliably predict effects on long-term growth, fruit development, or yield. Accordingly, these genes should be interpreted as indicators of regulatory potential rather than immediate breeding targets, particularly given evidence from rice and wheat that transcription factors often exert pleiotropic effects on growth, flowering, and yield [118,119].

#### 4.1.5. Signal Transduction and Post-Translational Regulators

Salinity responses in strawberry are associated with Ca^2+^ signaling, phosphorylation cascades, and ubiquitin-mediated protein turnover. Crizel et al. [113] characterized the FaCDPK family in octoploid strawberry as Ca^2+^-responsive kinases linking Ca^2+^ signatures to downstream stress responses; among the reported genes, *FaCDPK1* showed the strongest induction under salinity (≈15-fold). Responses to ABA and the ABA-biosynthesis inhibitor NDGA further support isoform-specific hormonal regulation rather than a simple ABA-independent salt model. Jiang et al. [114] provided complementary evidence for protein-level regulation by identifying 155 FaU-box (PUB) E3 ubiquitin ligases and profiling their expression during development and under abiotic stresses, including salt exposure at 200 mM NaCl. The study highlighted candidates including a salt-associated gene (*FaU-box3*) and additional members responsive across stress and hormonal contexts. Importantly, transient overexpression assays indicated that FaU-box127 is associated with changes in fruit composition traits, including soluble sugars and organic acids, emphasizing that post-translational regulators can affect both stress responses and fruit-quality processes [114].

From a breeding perspective, *CDPKs* and *U-box E3* ligases are unlikely to be immediate single-gene targets due to pleiotropic effects; accordingly, stress-responsive members such as *FaCDPK1* and salt-associated candidates such as *FaU-box3* are best prioritized for allele mining and QTL/GWAS colocalization, followed by validation under saline irrigation with yield and quality assessment [113,114].

#### 4.1.6. Structural and Epigenetic Contributors

Structural adaptations mediated by cell wall remodeling genes, including laccases (FvLAC family), contribute to cultivar-dependent stress endurance and are supported by expression analyses conducted directly in strawberry (Table 1) [115,116]. Kong et al. [115] also reported divergent functional roles among FvLAC isoforms, with FvLAC51 associated with enhanced tolerance and FvLAC24 and FvLAC32 linked to reduced performance, indicating specialization within this cell-wall remodeling family. Promoter analyses further suggest possible involvement of some *FvLAC* genes in fruit-related developmental processes, although their agronomic relevance under salinity remains to be established. Nevertheless, as observed in barley and maize, links between cell wall modification and yield under salinity remain largely indirect.

Similarly, epigenetic modifications, such as stress-associated DNA methylation changes, provide insight into transcriptional plasticity and stress memory in strawberry [117]. While these mechanisms are biologically compelling, their relevance to breeding depends on the stability and heritability of epigenetic states under agricultural conditions.

### 4.2. Breeding Implications

Overall, the reviewed evidence reveals a clear hierarchy in the breeding relevance of molecular mechanisms underlying salinity tolerance in strawberries. Genes directly controlling ion homeostasis—particularly members of the SOS and NHX families—emerge as the most credible near-term breeding targets, supported by sustained salinity experiments conducted in strawberry and by yield-validated examples from rice [96,97,98]. In contrast, genes involved in osmotic adjustment and oxidative stress regulation provide essential physiological support but lack consistent validation for fruit yield stability. Transcription factors and upstream signaling regulators, many of which have been characterized primarily through heterologous expression systems, should therefore be regarded as promising candidates whose breeding value will depend on strawberry-specific validation, careful regulatory tuning, and assessment of potential pleiotropic effects on growth, development, and fruit traits.

Despite progress in elucidating the molecular basis of salinity tolerance, translation of this knowledge into durable breeding outcomes remains uneven. Bridging this gap will require integrating mechanistic insights with long-term saline irrigation trials that extend to fruit yield and quality, together with genomic approaches that exploit natural allelic variation rather than relying solely on transgenic overexpression [121]. Such coordinated strategies will be essential for enhancing strawberry resilience under saline cultivation conditions.

## 5. Future Perspectives

Although substantial progress has been made in elucidating physiological responses, identifying stress-responsive genes, and evaluating biological mitigation strategies, a clear gap persists between current knowledge and its effective deployment in breeding programs and commercial production systems.

A central priority is the functional validation of candidate genes in relation to reproductive performance rather than vegetative tolerance alone. Numerous genes involved in ion homeostasis, osmotic adjustment, antioxidant defense, and transcriptional regulation have been identified; however, most have been characterized at early developmental stages or in heterologous systems, and rarely under field conditions. Future studies should prioritize strawberry-specific validation under prolonged salinity, extending evaluation to flowering, fruit set, yield stability, and fruit quality under realistic field conditions. In this context, genes directly regulating Na^+^ and Cl^−^ exclusion, compartmentation, and long-distance transport represent the most promising breeding targets, as they address the primary causes of yield loss while minimizing growth penalties.

The exploitation of natural allelic variation, rather than constitutive gene overexpression, should underpin future genetic improvement efforts. High-throughput phenotyping combined with genomic selection, marker-assisted breeding, and allele mining will be essential for identifying favorable variants that confer tolerance while preserving agronomic performance. CRISPR/Cas-based engineering may further accelerate the development of elite cultivars. Crop wild relatives, particularly salinity-tolerant accessions of *F. chiloensis*, remain an underutilized reservoir of adaptive traits. Their systematic integration through pre-breeding and advanced backcross populations will be critical for broadening the genetic base of cultivated strawberries, provided introgression is coupled with rigorous selection for agronomic and market traits.

In parallel, biological and agronomic interventions should be repositioned as complementary tools that enhance the expression of genetic tolerance rather than stand-alone mitigation measures. Biostimulants, small signaling molecules, and beneficial microorganisms consistently improve physiological resilience; however, evidence for their effects on yield and fruit quality remains limited. Future research should therefore focus on genotype × treatment interactions, enabling the co-development of tolerant cultivars and optimized management protocols. In this context, plant–microbe interactions represent a particularly promising yet underexploited research path. Arbuscular mycorrhizal fungi and plant growth–promoting rhizobacteria improve nutrient uptake, ion homeostasis, and root system functionality under saline conditions, and incorporating microbial responsiveness as a selection criterion could substantially enhance cultivar performance.

Additionally, advances in controlled-environment cultivation provide more opportunities to accelerate progress. Manipulation of light spectra can be used to refine stress exposure and improve salinity responses, thereby maintaining fruit quality and yield.

Overall, progress in this field will require a transition from stress tolerance screening to resilience-driven cultivar design. Successful strawberry improvement under salinity will depend on pyramiding ion-regulatory capacity, physiological plasticity, and yield stability, supported by targeted biological and agronomic interventions. The identification of cultivars capable of exploiting moderate salinity without compromising productivity, by achieving an optimal balance between mild salinity-induced improvements in fruit quality and yield penalties, could also become a specific breeding objective. By integrating molecular knowledge with long-term field validation and breeding-oriented selection, it will be possible to develop strawberry cultivars that are not only salt-tolerant but also productive and economically viable under saline cultivation conditions.

## 6. Conclusions

This review provides a framework for breeding strategies and other mitigation solutions aimed at enhancing strawberry resilience to saline environments and offers practical insights to support growers managing production in salt-affected regions. In this context, it synthesizes current knowledge of strawberry responses to salinity stress, with emphasis on physiological, biochemical, and molecular mechanisms. However, available evidence indicates that most of these responses have been characterized primarily at vegetative or early developmental stages, and their relevance to fruit yield and quality remains limited or context dependent.

Significant genotypic variation shapes the degree of salinity tolerance among strawberry cultivars and CWRs, and many genes have been identified as stress-related, highlighting the complexity of tolerance mechanisms and the need for careful interpretation of stress-response traits, although often without a clear impact on yield. Taken together, the available evidence indicates that although several approaches exist to mitigate salinity stress in strawberries and can improve plant performance and survival under saline conditions, these strategies do not necessarily guarantee stable or enhanced fruit yield. Overall, this synthesis underscores the distinction between mechanistic indicators of salinity response and traits with demonstrated agronomic relevance in strawberry production under saline conditions.

## Figures and Tables

**Table 1:** Genetic material with salinity tolerance in relation to yield reduction.

Genotype	Salinity Tolerance Level	Experimental Setup/Salinity Treatment	Key Physiological & Biochemical Traits	Yield & Survival Performance	References
Chilean strawberry genotypes ‘Bau’ (*F. chiloensis* ssp. *chiloensis* f. *chiloensis*)-cultivated and the wild ‘Cucao’ (*F. chiloensis* ssp. *chiloensis* f. *patagonica*)	Tolerant	The experiments were conducted in a polycarbonate greenhouse with natural light and temperature. Stress levels were 0, 30, and 60 mM NaCl solution (EC = 1.6, 3.4, and 5.7 dS m^−1^, respectively)	Leaf relative water content (LRWC), pigment concentrations, proline, malondialdehyde (MDA) concentration, total soluble sugars (TSS), and titratable acidity (TA)	No significant yield losses compared to “Camarosa” cultivar (25% yield loss at severe stress). Moreover, no differences were detected in fruit diameter (mm) or fruit length (mm)	[40]
*F. × ananassa* ‘Korona’	Relatively tolerant	Strawberry plants grown in a sandy medium and watered with solutions of moderate (ECe = 3.9 dS/m), and excessive (ECe = 7.5 dS/m) NaCl concentrations	Significant increase in reduced glutathione. Maintained high fruit quality and better taste under stress	Higher yield stability compared to the more sensitive cultivar ‘Elsanta’ under equivalent stress (fresh weight reduction of 26% versus 46%)	[33]
*F. × ananassa* ‘Elsanta’	Relatively tolerant	Strawberry plants grown in growth chamber in pots watered with 10, 20, or 40 mM NaCl	Low stomatal density/reduced transpiration/delay in the accumulation of Cl^−^ ions in the shoot	No significant yield reduction under all NaCl treatments	[43]
*F. × ananassa* ‘Albion’	Relatively tolerant	USDA-ARS U.S. Salinity Laboratory CA, using 12 raised beds to evaluate five strawberry cultivars under four salinity levels (0.7, 1.0, 1.5, and 2.5 dS m^−1^	Superior chloride exclusion from roots and petioles. High total soluble sugars (Brix%) regardless of salinity treatment	It exhibited the least mean relative reduction in fruit yield, marketable fruit size survival (94%) while maintaining its fruit taste compared to ‘San Andreas’, ‘Benicia’, ‘Ventana’, and ‘Monterey’	[17]
*F. × ananassa* ‘Rociera’ and ‘Camarosa’	‘Rociera’: relatively higher tolerance ‘Camarosa’: relatively lower tolerance	Greenhouse, NaCl solution with EC = 2 dS m^−1^; and (c) NaCl solution with EC = 4 dS m^−1^	Above-ground plant mass (AGPM), total plant fresh and dry biomass, root fresh and dry weight, length, weight, and diameter of each individual fruit, overall fruit production, and various antioxidants	‘Rociera’: reduction in the plant growth parameters, the number of leaves and plant water content under severe salinity conditions. ‘Camarosa’: low salinity tolerance index, low plant water content and growth parameters, but high fruit sucrose, fructose, glucose, and total sugar concentration, as well as sweetness index	[22]
*F. × ananassa* ‘Camarosa’	‘Camarosa’: tolerant	Greenhouse Seedlings grown in 9 L pots containing a mixture of soil and vermiculite Salt solution of 40 mmol/L NaCl in distilled water); L2 (stress level 2—salt solution of 80 mmol/L NaCl in distilled water) for a week	Yield of fruit, fresh biomass, and root biomass CO_2_ assimilation Content of sodium (Na) and chloride (Cl) in leaves Phenylalanine ammonia lyase (PAL), peroxidase (POD), and polyphenoloxidase (PPO) activity Total content and the content of reducing sugars Various antioxidants	Mild salt stress did not affect fruit yield The lower level of mild salt stress positively affected photosynthesis and fresh biomass Mild salt stress improved (a) the content of antioxidant compounds and sucrose in the fruit. (b) the content of anthocyanins and sucrose. Higher levels of salt stress induced root growth and the accumulation of phenolic compounds	[20]

**Table 2:** Functional categories of key genes involved in strawberry salinity and their roles in abiotic stress responses.

Functional Category	Genes/Gene Families	Functional Relevance in Abiotic Stress	Reference
Ion homeostasis and transport	*SOS1*, *SOS2*, *SOS3*, *NHX1*, *NHX2*, *CLC_C*, *CLC_G*, *KUP6*, *KUP7*, *SLAH3*, *ALMT12*, ABCB/C/G transporters	Regulation of Na^+^ and Cl^−^ transport, vacuolar sequestration, and K^+^ balance, contributing to salt tolerance and ionic homeostasis	[98,99,100]
Osmotic adjustment and water balance	Osmotin (*PR-5*), *LEA3*, *P5CS1*, *P5CS2*, *ProDH2*, *P5CDH*, *NCED1*, *NCED3*, *SnRK2.4*, *SnRK2.6*, Aquaporins (*PIP*, *TIP*, *NIP*, *SIP*)	Maintenance of cellular turgor and water potential through compatible solute accumulation, ABA-mediated regulation, and water transport	[98,100,101,102,103,104,105]
Oxidative stress regulation and redox balance	*FaCSDs*, *FaFSDs*, *FaMSDs* (*FaMSD5*), *CAT*, *POD*, *APX*, *FaG6PDH1–FaG6PDH19*, *GSTU5*, *CHS*, *CHI*, *F3H*, *DFR*, *ANS*	Detoxification of reactive oxygen species (ROS), maintenance of redox homeostasis, and antioxidant protection under stress	[99,106,107,108,109]
Transcriptional control of stress responses	*FvNAC01–37*, *FaNAC2*, *FvNAC29*, *FaTINY2* (AP2/ERF), *FvMYB24*, *FvMYB82*, *MYB1*, *MYB5*, *MYB30*, *MYB33*, *MYB101*, *MYB108*, *FaZAT10* (C2H2-ZFP), *FvGRF1–10*, *FaTEDT1L* (HD-ZIP IV)	Regulation of downstream gene networks controlling ion homeostasis, osmotic adjustment, oxidative stress responses, growth–stress balance, and ABA signaling	[99,100,103,104,105,108,109,110,111,112]
Signal transduction and post-translational regulation	*FaCDPK1–FaCDPK11*, RBOH (CDPK-linked), FaU-box E3 ligases (*FaU-box98*, *FaU-box127*, *FaU-box136*, etc.)	Calcium- and ROS-mediated signaling and ubiquitin-dependent protein turnover modulating stress perception and response intensity	[113,114]
Cell wall remodeling and structural maintenance	*FvLAC1–57* (notably *FvLAC24*, *FvLAC32*, *FvLAC51*), *CesA*, *XHT*, *EXP*, *PL*, lignin- and cellulose-associated DEGs	Modification of cell wall composition and mechanical properties contributing to stress endurance and cultivar-dependent tolerance	[115,116]
Epigenetic regulation	Stress-associated DMRs (differentially methylated regions)	DNA methylation changes affect transcriptional plasticity and stress memory	[117]

## Data Availability

No new data were created or analyzed in this study.

## Supplementary Materials

The following supporting information can be downloaded at: https://www.mdpi.com/article/10.3390/plants15030432/s1, Table S1: Responses of strawberry cultivars to salt stress at vegetative/no fruiting stage; Table S2: Studies referring to small signaling molecules and biostimulants involved in mitigating salinity stress, including the effects of supplemental lighting, microorganisms, and plant–microbe interactions under saline conditions; Table S3: Experimental settings, plant developmental stages, growth conditions, salinity stress protocols and parameters identified for all studies summarized in Table 2.
