# Supplementary material for: Salinity Stress in Strawberry (Fragaria × ananassa Duch.): Biological Intervention Strategies and Breeding Approaches for Salt-Tolerant Cultivars
Source: Plants (Basel). 2026 Jan 30;15(3):432. doi: 10.3390/plants15030432 (PMC12899836; doi:10.3390/plants15030432)
Supplement: Supplementary file 1 [file plants-15-00432-s001.zip › Supplementary Table S3.pdf]

Supplementary Table S3: Experimental settings, plant developmental stages, growth conditions, salinity stress protocols and parameters identified for all studies summarized in Table 2.

Study	Experimental Setting	Plant Material & developmental stage	Growth Conditions (substrate, environment)	Salinity Stress Level & Duration	Parameters Measured & Analyzed
[106]	Plastic pots & Orchard	(*F. × ananassa* 'Benihoppe') seedlings. For salinity; 5 fruit stages (green to full red)	Pots; Substrate: vermiculite, perlite, and peat soil (1:1:4).	200 mM NaCl for 24 h.	Gene identification, qRT-PCR in roots/leaves, and cis-acting elements.
[115]	Phytotron (Growth Chamber)	*F. vesca* 10-week-old seedlings.	Substrate: soil and vermiculite (1:4); 16h light/22°C.	300 mM NaCl root irrigation; 0–12 h time-course.	RT-qPCR expression, transgenic yeast stress tolerance, and subcellular localization.
[110]	Growth chamber & Greenhouse	*F. vesca* 100-day-old plants; tissues include leaf, stem, root, flower, and full red fruit.	24±2℃ under a 14 h/10 h light/dark photoperiod with 80% relative humidity.	100 mM NaCl irrigation; sampled from 2h to 24 days.	Spatial expression profiling and qRT-PCR response to abiotic/biotic stress.
[103]	Plant culture room	*F. × ananassa* 'Benihoppe' 3-month-old tissue cultured seedlings. Transgenic *N. benthamiana*: 13-day-old (seeds) and 40-day-old (seedlings)	Soil substrate; controlled temperature and light	200 mM NaCl (strawberry); up to 300 mM (transgenic N. benthamiana).	GUS activity, proline metabolism, antioxidant enzymes (CAT, POD, SOD), and water loss.

[104]	Growth chamber	2-week-old *F. vesca* seedlings.	Substrate: nutrition soil and vermiculite (2:1); 16h light/22°C.	200 mM NaCl irrigation for 7 days.	Survival rate, physiological markers (chlorophyll, proline, MDA, ROS), and enzyme activities.
[105]	Growth chamber	Strawberry (*F.* × *ananassa*) heterologous expression in *Arabidopsis thaliana*	2-week seedlings (strawberry); cotyledon-exposed stage (Arabidopsis). Substrate: soil, vermiculite, perlite (2:1:1).	200 mM NaCl irrigation for 7 days.	Survival %, physiological indicators (MDA, H_2O_2, chlorophyll), and downstream gene expression.
[116]	Greenhouse	Different varieties of strawberry (*F.* × *ananassa* Duch.*)* seedlings	6–7 leaf stage. Substrate: peat moss and perlite (2:1); 16h light/25°C.	50, 100, 150, 200 mM NaCl for 50 days (weekly irrigation).	Salt damage index (SDI), biomass, transcriptomic (RNA-seq), and metabolomic profiles.
[99]	Growth chamber & Greenhouse	*F. vesca* 'Hawaii 4' heterologous expression in *Arabidopsis thaliana*/ 10-day-old seedlings.	Substrate: organic soil and sand (3:1); 16h light/22°C.	100, 150, 200 mM NaCl (agar) or 200 mM (soil) for 7d to 4wks.	Germination rate, root length, fresh weight, chlorophyll, antioxidant activities, and Y1H.

[112]	Incubator	*F. vesca* seedlings/ *A. thaliana* transformed seedlings	Seedling stage (*F. vesca*); cotyledon-exposed (Arabidopsis). Substrate: soil and vermiculite (2:1).	200 mM NaCl (strawberry 12h; Arabidopsis 7 days).	Germination/survival rate, MDA, proline, enzymes (CAT, POD, SOD), and EL.
[109]	Controlled environment	*F.* × *ananassa* Duch. 'Petaluma' and 'Cabrillo' cultivars at three-leaf stage.	Substrate: soil, peat, and perlite (1:1:1); 16/8 h light/dark, 23/18 °C day/night temperatures, 65% relative humidity.	150 and 300 mM NaCl for 10 days (gradual 3-day increase).	Growth parameters, photosynthetic pigments, oxidative markers, and anthocyanin content.
[117]	Plant growth chambers/In vitro (MS plates)	One-month-old seedlings *F. vesca* cv. Reine des Vallées (RV)	Agarose plates; Media: 50% MS + 30% sucrose + 2% phytagel; 16h light/24°C.	100 mM NaCl for 2 weeks (with recovery days).	Epigenetic profiling; WGBS (global DNA methylation), DMR density, and RNA-seq.
[100]	Growth chamber	(*F.* × *ananassa* Duch. cv. Toyonoka) / *Arabidopsis thaliana* 14-day-old seedlings.	Substrate: 1/2 MS medium (in vitro) and potting soil.	100–150 mM NaCl for 10 to 14 days.	Survival rate, root length, water loss, qPCR, and transactivation.
[107]	Greenhouse	*F.* × *ananassa* cv. Benihoppe/ Seedlings and 6 fruit stages (green to red).	Standard greenhouse cultivation.	100 mM NaCl for 72 hours.	Identification, synteny, cis-element analysis, and qPCR expression time-course.

[113]	Greenhouse	*F.* × *ananassa* cv. Camarosa; Ripe fruits and 6 fruit developmental stages (SG to FR).	Pots; Substrate: Ultisoil and vermiculite (3:1).	80 mM NaCl (SS treatment) via spray.	RT-qPCR (fruit and stress), Ka/Ks evolutionary analysis, and protein-protein interactome.
[108]	Growth chamber/ tissue culture	Strawberry 'Benihoppe' (*F.* × *ananassa* Duch.) Seedlings; 6 fruit developmental stages (LG to FR)	Substrate: Hoagland nutrient solution.	200 mM NaCl for 24 hours.	Gene identification, RNA-seq mining, nucleus localization, yeast transactivation, and qPCR.
[114]	Greenhouse	5 fruit developmental stages (SG to FR)	Standard cultivation: 14h light/22°C.	200 mM NaCl for 24 hours.	RNA-seq, GO enrichment, interaction network, and transient overexpression (FaU-box127).
[101]	Growth chamber-controlled temperature and photoperiod	Suspension cultures of tobacco (*Nicotiana tabacum* var Wisconsin 38)	Liquid media	171 mM (S-10 cells) to 428 mM (S-25 cells).	Osmotin cDNA isolation, mRNA induction/stability, and endogenous ABA levels.

[98]	Greenhouse lysimeters	*F. vesca*,4-week-old plantlets (vegetative stage).	Potted plants, Substrate: sand tanks; 23–29°C.	ECiw 8.0 dS m^{-1} for 4 weeks ~85–90 mM NaCl (controlled irrigation, gradual 3-day increase).	Shoot/root biomass, salt tolerance (ST) index, tissue ions (Na, Cl, K), and gas exchange.
[111]	Growth room	*F. vesca*,4-month-old seedlings.	Potted plants; substrate: soil; 13h light/22°C.	400 mM NaCl (foliar spray) for 12 hours.	qRT-PCR (organs/stress), cis-acting elements, and duplication events analysis.
[102]	Growth chamber/In vitro	Transgenic Strawberry / Leaf discs and plantlets.	Media: MS salts + B5 vitamins + 2% glucose.	0 to 200 mM NaCl for 2 weeks.	Transgene integration, chlorophyll, protein, proline, RWC, and biomass.
