# Supplementary material for: Salinity Stress in Strawberry (Fragaria × ananassa Duch.): Biological Intervention Strategies and Breeding Approaches for Salt-Tolerant Cultivars
Source: Plants (Basel). 2026 Jan 30;15(3):432. doi: 10.3390/plants15030432 (PMC12899836; doi:10.3390/plants15030432)
Supplement: Supplementary file 1 [file plants-15-00432-s001.zip › Supplementary Table S1.pdf]

Supplementary Table S1: Responses of strawberry cultivars to salt stress at vegetative/no fruiting stage

Study	*F.* × *ananassa* cultivars	Primary Traits Studied	Duration of the experiment	Reason for No Yield Data
**[25]**	'Fortuna', 'Festival' (no significant differences)	Chlorophyll, Proline, Antioxidant activity	3 weeks	*In vitro* regeneration
**[52]**	'Albion'	Shoot number, POD/CAT activity	30 days	Developing salinity-tolerant clones through in vitro mutagenesis (gamma irradiation)
**[42]**	'Camarosa', 'Sweet Charlie'	N and P concentrations in the leaves and roots	6 months	Focus on nutrient assimilation.
**[44-46]**	'Camarosa' is referenced in all papers, whereas 'Chandler' and 'Tioga' are each reported in a single study.	Amino acids, Micronutrients	80-182 days	Focus on osmotic adjustment.
**[47]**	'Camarosa', 'Tioga', (more tolerant) and 'Chandler' (less tolerant)	Antioxidant activities, electrolyte leakage, protein soluble levels	30 days	Focus on biochemical properties.
**[53]**	'Tioga' and 'Rapella' more tolerant than the other tested	Morphological traits,MDA content, K/Na and Ca/Na ratios	15 days	Focus on vegetative indices (number of leaves, the fresh weight of individual organs, total plant weight).
**[23]**	'Kardistan' (more tolerant), 'Queen Eliza' (susceptible)	Antioxidant activity, stomatal conductance, RWC	60 days	Focus on vegetative, physiological and biochemical parameters at early stages
