# Supplementary material for: Salinity Stress in Strawberry (Fragaria × ananassa Duch.): Biological Intervention Strategies and Breeding Approaches for Salt-Tolerant Cultivars
Source: Plants (Basel). 2026 Jan 30;15(3):432. doi: 10.3390/plants15030432 (PMC12899836; doi:10.3390/plants15030432)
Supplement: Supplementary file 1 [file plants-15-00432-s001.zip › Supplementary Table S2.pdf]

Supplementary Table S2. Studies referring to small signaling molecules and biostimulants involved in mitigating salinity stress, including the effects of supplemental lighting, microorganisms, and plant–microbe interactions under saline conditions.

Study	Experimental Setting	Plant Material & Developmental Stage	Growth Conditions	Salinity stress level & Duration	Parameters Analyses
**Studies refer to small signaling molecules and biostimulants in mitigating salinity stress**					
[62]	Greenhouse pot experiment; root application of ALA	Strawberry (*Fragaria* × *ananassa* Duch., cv. Benihonpe); four fully expanded leaf stage	Plastic pots (8 kg garden soil + 7.5% mature cattle manure); standard greenhouse management	Salinity stress mentioned but stress level and duration not specified	Leaf gas exchange; chlorophyll fluorescence; PSII and PSI activity; electron transport characteristics
[63]	Controlled growth chamber; root irrigation with ALA under osmotic and salt stress	Strawberry (*Fragaria* × *ananassa* cv. Benihoppe); five-leaf-old (osmotic stress) and seven-leaf-old (salt stress) plants	Individual cups with peat: vermiculite: perlite (4:2:1); irrigated with 1/2 Hoagland solution every 3 days	100 mM NaCl for 6 days (salt stress); 20% PEG for up to 6 days (osmotic stress)	Na^+ distribution (roots/leaves); expression of Na^+ transporter genes (*SOS1*, *NHX1*, *HKT1*); H_2O_2 content; root physiological traits; biochemical assays; RNA-seq
[64]	Controlled growth chamber; root irrigation with ALA under osmotic stress	Strawberry (*Fragaria* × *ananassa* cv. Benihoppe); five-leaf-old plants	Individual cups with peat:vermiculite:perlite mixture; irrigated with 1/2 Hoagland solution every 3 days	Osmotic stress induced by 20% PEG for up to 6 days (sampling at 2, 4, and 6 days)	Physiological traits; antioxidant enzyme activities; biochemical parameters; RNA-seq analysis
[68]	Greenhouse pot experiment; foliar application of melatonin under salinity stress	Strawberry (*Fragaria* × *ananassa* cv. Camarosa); five fully expanded leaf stage	7-kg pots with coconut coir:perlite (1:1); daily irrigation with full-strength Hoagland solution	0, 40, and 80 mM NaCl for 58 days	Fruit yield and quality traits; leaf photosynthetic pigments; macronutrient concentrations; antioxidant enzyme activities; oxidative stress markers; ABA content; fruit biochemical traits
[70]	Greenhouse pot experiment; exogenous salicylic acid application under salinity stress	Strawberry (*Fragaria* × *ananassa*); developmental stage not specified	Potted plants grown with complete nutrient solution	0 or 35 mM NaCl	Growth parameters; chlorophyll content; leaf relative water content (LRWC); electrolyte leakage; mineral nutrient concentrations in leaves and roots

[71]	Greenhouse pot experiment; foliar salicylic acid application with different timing relative to salinity stress	Strawberry (*Fragaria* × *ananassa* cv. Selva); four–five fully expanded leaf stage	3-L pots with peat:perlite (1:1); daily fertigation with modified Hoagland solution	40 mM NaCl applied from day 14 and maintained for ~2 months	Mineral nutrient concentrations in roots; growth- and nutrition-related physiological responses
[72]	Greenhouse pot experiment; foliar salicylic acid pretreatment under salinity stress	Strawberry (*Fragaria* × *ananassa* cv. Gaviota); similar size and developmental stage	Plastic pots with perlite:coco peat (1:1); irrigated with half-strength Hoagland solution; greenhouse near Malekan, NW Iran; 22–26/16–18 °C day/night, 65–68% RH, 400–450 μmol m^{-2} s^{-1} light	50 mM NaCl applied in nutrient solution 7 days after SA pretreatment; 10 days of stress; NaCl added gradually in two steps; solution renewed every 4 days; pH 6	Growth traits; PSII performance; soluble sugar content; antioxidant enzyme activity; phenolic compounds; oxidative membrane damage indicators
[73]	Field pot experiment (two seasons); foliar salicylic acid application under salinity stress	Strawberry (*Fragaria* × *ananassa* cv. Camarosa); four–five leaf stage	20 cm plastic pots with perlite:peat moss (1:1); Hoagland solution irrigation every 48 h	0, 20, and 40 mM NaCl applied via irrigation (stress duration: growing season)	Growth traits; Salt Tolerance Index (STI); antioxidant enzymes; proline content; fruit yield & quality traits
[80]	Greenhouse experiment (two seasons); factorial design with root applications of salinity and humic acid (HA) treatments	Strawberry (*Fragaria* × *ananassa* cvs. Kurdistan and Paros); 5–6 fully expanded leaf stage	Coco peat–perlite substrate; greenhouse conditions 14–36 °C, 60–70% RH, PAR 650–1200 μmol m^{-2} s^{-1}; daily 200 mL nutrient solution (EC 0.8 dS m^{-1}, pH 6–6.5) until growth initiation	0 or 50 mM NaCl (weekly 500 mL water to prevent NaCl accumulation); in Mar–Oct 2014, & Mar–Jun 2015)	Vegetative growth traits; relative water content; membrane stability; chlorophyll content; Na^{+} and K^{+} concentrations; oxidative stress markers; proline and soluble carbohydrates; antioxidant enzyme activities; salt tolerance index; total biomass and fruit yield
[81]	Greenhouse pot experiment; foliar acetic acid application with different timing relative to salinity stress	Strawberry (*Fragaria* × *ananassa* cv. Paros); 4–5 fully expanded leaf stage	Pots with coco-peat:perlite (1:1); fertigated with Hoagland solution	0 mM and 40 mM NaCl (stress duration not explicitly stated)	Growth traits; chlorophyll content; fruit traits; proline and soluble sugar content; fruit antioxidant capacity
[82]	Greenhouse experiment; nutrient solution application of methyl jasmonate (MeJA)	Strawberry (*Fragaria* × *ananassa* cv. Paros); 5–6 fully expanded leaf stage	Perlite–coco peat substrate; controlled greenhouse conditions	50 mM NaCl (stress duration not specified; leaf sampling at 1, 24, 48 h after treatment)	Physiological traits; expression of salinity-related genes

	and silicon nanoparticles (Si NPs) under salinity stress				
**Studies refer to supplemental light application**					
[91]	Greenhouse experiment; salinity and alkalinity treatments under 5 light levels	Strawberry (*Fragaria* × *ananassa* Duch., cv. Camarosa); fully developed leaves (age of 60 days after planting)	Temperature 25/15 ± 2 °C (day/night), 11/13 h light/dark photoperiod, 50 ± 10% RH. Plants grown in 4 L pots with cocopeat:perlite (70:30). Irrigation with Morgan nutrient solution (EC 1.4 dS m^{-1}, pH 6.5). LED lights applied at different spectral qualities with PPFD ≈ 1000 µmol m^{-2} s^{-1}, 30 cm above plants.	80 mM NaCl (salinity), 40 mM $NaHCO_3$ (alkslinity) gradual stress starting at 40 mM NaCl / 20 mM $NaHCO_3$ for 60 days	Chlorophyll fluorescence and leaf gas exchange parameters
[93]	Greenhouse experiment; four salinity and alkalinity treatments under 6 light levels	Strawberry (*Fragaria* × *ananassa* Duch., cv. Sabrina); fully developed leaves and fruits	Maintained at 25/15 ± 2 °C (day/night), 11/13 h light/dark photoperiod, relative humidity 50 ± 10%. Plants grown in 4 L pots with cocopeat:perlite (70:30). Fertigation with Morgan nutrient solution (EC 1.4 dS/m, pH 6.5). LED lights installed above plants with uniform PPFD of 200 µmol m^{-2} s^{-1} for light treatments.	80 mM NaCl (salinity), 40 mM $NaHCO_3$ (alkalinity), and combined 80 mM NaCl + 40 mM $NaHCO_3$. Stress applied starting 20 days after planting, continued for 60 days (total experiment duration 80 days).	Vegetative growth; yield and fruit quality; leaf osmotic characteristics; enzyme activity; mineral analysis
[92]	Greenhouse experiment; four salinity and alkalinity treatments under 6 light levels	Strawberry (*Fragaria* × *ananassa* Duch., cv. Camarosa; fully developed leaves and fruits	Temperature 25/15 ± 2 °C (day/night), photoperiod 13/11 h light/dark, relative humidity 50 ± 10%. Maximum daily light intensity 1085 µmol m^{-2} s^{-1} (LED + ambient). Plants grown in 4 L pots with cocopeat:perlite (70:30). Fertigation applied with Morgan nutrient solution (EC 1.4 dS/m, pH 6.5). LED lights mounted 30 cm above plants with PPFD maintained at 200 µmol m^{-2} s^{-1} for spectral treatments.	80 mM NaCl (salinity) and 40 mM $NaHCO_3$ (alkalinity). Solutions applied 100 mL per pot every three days. Stress treatments applied 20 days after planting and duration of stress until end of experiment	Vegetative growth; yield traits and fruit quality; mineral analysis

**Studies refer to microorganisms and plant-microbe interactions under salinity**					
[86]	Greenhouse pot experiment; inoculation with Gigaspora margarita under salinity stress	Strawberry (*Fragaria* × *ananassa* cv. Tochiotome); two-month-old plants	Fertilized pots; controlled greenhouse conditions	200 mM NaCl for 12 days	Shoot and root biomass; chlorophyll content; leaf browning; Na^+ accumulation and Na^+/K^+ ratio in roots and shoots; SEM-EDX analysis of Na^+ localization; root cellulose and lignin content
[89]	Controlled-environment chamber experiment; PGPR inoculation under salinity stress and varying CO_2/temperature regimes	Strawberry (*Fragaria vesca* var. Fortuna); cold-stored bare-root plants, establishment for 15 days before treatments	1.5 L pots with sterilized sand:organic substrate (4:1); controlled temperature and CO_2	0 or 85 mM NaCl applied via 25-min pot immersion; stress duration 30 days	Shoot and root biomass; photosynthetic performance
[94]	Controlled experiment; root/application of hydrogen-rich water (HRW) under salinity stress	Strawberry (*Fragaria* × *ananassa* cv. Benihoppe); seedling stage	Growth conditions not fully specified; presumably controlled environment	100 mM NaCl (Na) and 100 mM NaCl +100% HRW (Na_HRW); 14days with gradual mitigation	Growth traits; K^+ and Na^+ content; antioxidant activity; metabolite profiling; transcriptomic and metabolomic analyses
[75]	Greenhouse pot experiment; foliar GABA application under salinity stress	Strawberry (*Fragaria* × *ananassa* cv. Benihoppe); seedling stage, uniform plants selected before treatments	Pots under greenhouse conditions	0 or 200 mM NaCl applied every 3 days for 21 days	Photosynthetic traits; osmolyte accumulation; antioxidant capacity; ROS and MDA levels; transcriptomic analysis
[7]	Greenhouse pot experiment; foliar GABA application and salinity treatments	Strawberry (*Fragaria* × *ananassa* cv. Aromas); well-grown, uniform plantlets with 5–6 fully expanded leaves	7-L plastic pots with 1:1 coco peat:perlite; greenhouse: 20–23 °C, 65–75% RH, PAR 700–1200 μmol m^{-2} s^{-1}; 2.7 plants/m^2; 500 mL half-strength Hoagland per plant	3 and 5 dS m^{-1} EC via NaCl	H_2O_2, MDA, proline, MSI, TSP, antioxidant enzymes; chlorophyll content, net photosynthetic rate, fruit weight; semi-quantitative RT-PCR
